# Trichoderma species occurring on wood with decay symptoms in mountain forests in Central Europe: genetic and enzymatic characterization

**DOI:** 10.1007/s13353-015-0326-1

**Published:** 2015-11-19

**Authors:** Lidia Błaszczyk, Judyta Strakowska, Jerzy Chełkowski, Agnieszka Gąbka-Buszek, Joanna Kaczmarek

**Affiliations:** Institute of Plant Genetics, Polish Academy of Sciences, Strzeszyńska 34, 60-479 Poznań, Poland

**Keywords:** Cellulases, Molecular identification, ITS1, ITS2 rRNA, *tef1*, Xylanases

## Abstract

The aim of this study was to explore the species diversity of *Trichoderma* obtained from samples of wood collected in the forests of the Gorce Mountains (location A), Karkonosze Mountains (location B) and Tatra Mountains (location C) in Central Europe and to examine the cellulolytic and xylanolytic activity of these species as an expression of their probable role in wood decay processes. The present study has led to the identification of the following species and species complex: *Trichoderma atroviride* P. Karst., *Trichoderma citrinoviride* Bissett, *Trichoderma cremeum* P. Chaverri & Samuels, *Trichoderma gamsii* Samuels & Druzhin., *Trichoderma harzianum* complex, *Trichoderma koningii* Oudem., *Trichoderma koningiopsis* Samuels, C. Suárez & H.C. Evans, *Trichoderma longibrachiatum* Rifai, *Trichoderma longipile* Bissett, *Trichoderma* sp. (*Hypocrea parapilulifera* B.S. Lu, Druzhin. & Samuels), *Trichoderma viride* Schumach. and *Trichoderma viridescens* complex. Among them, *T. viride* was observed as the most abundant species (53 % of all isolates) in all the investigated locations. The Shannon’s biodiversity index (H), evenness (E), and the Simpson’s biodiversity index (D) calculations for each location showed that the highest species diversity and evenness were recorded for location A—Gorce Mountains (H′ = 1.71, *E* = 0.82, *D* = 0.79). The preliminary screening of 119 *Trichoderma* strains for cellulolytic and xylanolytic activity showed the real potential of all *Trichoderma* species originating from wood with decay symptoms to produce cellulases and xylanases—the key enzymes in plant cell wall degradation.

**Electronic supplementary material:**

The online version of this article (doi:10.1007/s13353-015-0326-1) contains supplementary material, which is available to authorized users.

## Introduction

Species of the ascomycete genus *Trichoderma* (teleomorph *Hypocrea*) are found in many ecosystems, but the most common and natural habitat of these fungi is known to be soil (Druzhinina et al. 2011). The occurrence of *Trichoderma* spp. in various soils, such as agricultural, forest, prairie, desert or salt marsh, of all climatic zones, has been the subject of several investigations (Druzhinina et al. 2011; Kubicek et al. 2008). Recent studies based on hight-throughput sequencing (Buee et al. 2009; Lim et al. 2010) and metagenomic analysis (Friedl and Druzhinina 2012; Hagn et al. 2007; Meincke et al. 2010) have revealed that soil is colonized by only a relatively small portion of *Hypocrea/Trichoderma* species, notably *Trichoderma asperellum* Samuels, Lieckf. & Nirenberg*, Trichoderma harzianum* complex*, Trichoderma hamatum* (Bonord.) Bainier*, Trichoderma atoviride* P. Karst.*, Trichoderma virens* (J.H. Mill., Giddens & A.A. Foster) Arx*, Trichoderma lonibrachiatum* Rifai*, Trichoderma gamsii* Samuels & Druzhin*., Trichoderma citinoviride* Bissett*, Trichoderma koningiopsis* Samuels, C. Suárez & H.C. Evans*, Trichoderma spirale* Bissett and *Trichoderma koningii* Oudem., which are known to generally have outstandingly high opportunistic potential. These species succeed in various heterotrophic interactions exhibiting saprotrophic and mycoparasitic (necrotrophic hyperparasitism, mycotrophy) lifestyles (Druzhinina et al. 2011).

As saprotrophs, *Trichoderma* have been considered to make a contribution to the degradation of plant debris, wood and bark (Klein and Eveleigh 1998). Plant biomass is mainly composed of cellulose (insoluble fibers of *β*-1,4-glucan), hemicellulose (xylans, mannans) and lignin (a complex polyphenolic structure), which together with proteins, fats, waxes, terpenes, phenols, alcohols and alkanes form the complex and rigid structure of the plant cell walls. Degradation of plant cell wall biopolymers is a complex process, which requires the synergistic action of a large number of extracellular enzymes. Several *Trichoderma* species, especially *Trichoderma reesei*, are known to be producers of cellulolytic and hemicellulolytic enzymes (Druzhinina et al. 2010; Gusakov 2011; Kubicek 2013). However, *Trichoderma* species are not able to decompose non-decayed wood lignocelluloses (Fukasawa et al. 2005, 2011). The utilization of cellulose and hemicellulose by *Trichoderma* spp. is possible only after preliminary depolymerization and mineralization of lignin by Basidio- and Ascomycetous white rot fungi (Aro et al. 2005; Fuksawa et al. 2005; 2011). *Trichoderma* spp. are perceived to interfere antagonistically with the primary wood decomposers, either via competition for nutrients and space substrate colonization or by using mechanisms of antibiosis and mycoparasitism (Jaklitsch 2009; Rossman 1996). A comparative analysis of the genome, secretome and transcriptome inventory of *T. atroviride*, *T. virens* and *Trichoderma reesei* indicated that mycoparasitism is the initial (ancestral) lifestyle of *Trichoderma* (Atanasova et al. 2013; Druzhinina et al.; 2012; Kubicek et al. 2011) whereas saprophytism on pre-degraded wood is suggested to be the result of the subsequent and alternative nutrient specialization of this fungus (Atanasova et al. 2013; Druzhinina et al. 2012; Kubicek et al. 2011). This hypothesis has been applied in particular to the best known saprotrophic *T. reesei* species, i.e. wild-type isolate QM 6a, which is extremely specialized in the degradation of plant cell wall cellulose and hemicellulose (Druzhinina et al. 2010; Kubicek 2013). It is worth noting that *T. reesei* has very rarely been detected in the natural environment. Only its teleomorphic stages (*Hypocrea jecorina*) and associated *Trichoderma parareesei* nom. prov. have been found worldwide in pantropical climatic zones (Druzhinina et al. 2010; Lieckfeld et al. 2000). Among the other *Trichoderma* species, the ability to produce the cellulase and hemicellulase complex has been stated for several strains of *T. harzianum* complex, *T. koningii*, *T. longibrachiatum*, *T. viride, T. virens* and *T. citrinoviride* (Strakowska et al. 2014). These species are known to have a pan-global distribution, although some of them occur frequently in temperate regions (Kubicek et al. 2008; Migheli et al. 2009). In our previous study on the occurrence and diversity of *Trichoderma* in Poland (Central Europe), five of the above-mentioned species (*T. harzianum* complex*, T. koningii, T. longibrachiatum, T. viride* and *T. citrinoviride*) were also isolated, mostly from pieces of decaying wood (Błaszczyk et al. 2011). Furthermore, decaying wood was found to be one of the most diverse habitats (nine species) from which the most isolates (75 isolates) were collected. Therefore, the objective of the present research was to explore the species diversity of *Trichoderma* obtained from samples of wood collected in the forests of the Gorce Mountains (location A), Karkonosze Mountains (location B) and Tatra Mountains (location C) in Central Europe and to examine the cellulolytic and xylanolytic activities of these species as an expression of their probable role in wood decay processes.

## Materials and methods

### Fungal collection

The 119 *Trichoderma* strains investigated in this study were obtained from samples of decaying wood collected in the forests of the Tatra Mountains, Gorce Mountains and Karkonosze Mountains, located in Central Europe in Poland (Table 1). Fifteen *Trichoderma* strains had previously been identified at the species level by Jeleń et al. (2014). One hundred and four *Trichoderma* isolates were described in this paper. All the studied *Trichoderma* strains are deposited in the collection of the Institute of Plant Genetics, Polish Academy of Science, Poznań, Poland, and are available to the scientific community.

**Table 1:** *Trichoderma* isolates originated from decaying wood in forests mountains in Poland

Species	Culture/strain code	NCBI GenBank accession No.^a^	
		ITS	*tef1*
Location^b^ A			
*T. atroviride*	AN497	JX184119	JX184096
*T. citrinoviride*	AN393 ^c^, AN490, AN710	JX184109	JX184086
*T. cremeum*	AN392	JX184117	JX184094
*T. harzianum* complex: (*T. harzianum*)	AN394	JX184113	JX184090
*T. longibrachiatum*	AN488	JX184118	JX184095
*T. koningii*	AN398, AN399	JX184126	JX184106
*T. paraviridescens*	AN387, AN405, AN492, AN494, AN709	JX184127	JX184103
*T. trixiae*	AN388	JX184128	JX184104
*T. viride*	AN397, AN402, AN493, AN496	JX184121	JX184098
	AN389, AN395, AN401	JX184122	JX184099
	AN390, AN487	JX184123	JX184101
Location B			
*T. citrinoviride*	AN499, AN500	JX184109	JX184086
*T. atroviride*	AN240	JX184119	JX184096
*T. koningiopsis*	AN251	HQ292939	HQ292993
*T. viridescens* complex: (*T. paraviridescens*)	AN241, AN243, AN245, AN609	JX184127	JX184103
(*T. trixiae*)	AN248	JX184128	JX184104
(*T. viridarium*)	AN605	JX184125	JX184102
*T. viride*	AN242, AN250, AN255, AN604	JX184121	JX184098
	AN244, AN249, AN810, AN813, AN826	JX184122	JX184099
	AN247, AN252, AN253, AN802, AN806, AN814, AN827	JX184123	JX184101
Location C			
*T. atroviride*	AN705	JX184119	JX184097
*T. citrinoviride*	AN303b, AN477	JX184109	JX184086
*T. gamsii*	AN327, AN385	JX184130	JX184107
*T. harzianum* complex: (*T. harzianum*)	AN312, AN360, AN367,AN369, AN373, AN381, AN415, AN479,	JX184113	JX184090
	AN349, AN480	JX184111	JX184089
(*T. atrobrunneum*)	AN364, AN699, AN370, AN704, AN706	JX184112	JX184088
*T. longipile*	AN359, AN414	JX184115	JX184091
*T. viridescens* complex: (*T. paraviridescens*)	AN322, AN323, AN328, AN334, AN702	JX184127	JX184103
(*T. trixiae*)	AN308, AN366, AN416	JX184128	JX184104
*T. viride*	AN310, AN330, AN350, AN332, AN340, AN357, AN356, AN358, AN361, AN378, AN384, AN419, AN421, AN478	JX184123	JX184101
	AN315, AN320, AN351, AN352, AN355, AN354, AN371, AN374, AN376, AN377, AN379, AN382, AN383, AN472, AN474, AN475, AN485, AN484, AN687, AN690, AN701	JX184121	JX184098
	AN346	JX184124	JX184100
	AN347, AN420	JX184122	JX184099
*Trichoderma sp.*	AN471	JX184114	JX184093

^a^the same NCBI GenBank Accession number assigned to the isolates possessing identical alleles in the locus ITS or *tef1*

^b^Location A—Gorce Mountains, location B—Karkonosze Mountains, location C—Tatra Mountains

^c^underline indicates the isolates identified previously at the species level by a combination of morphological and molecular analyses by Jeleń et al. (2014)

### Study site, strain isolation and maintenance

**Fig. 1:**
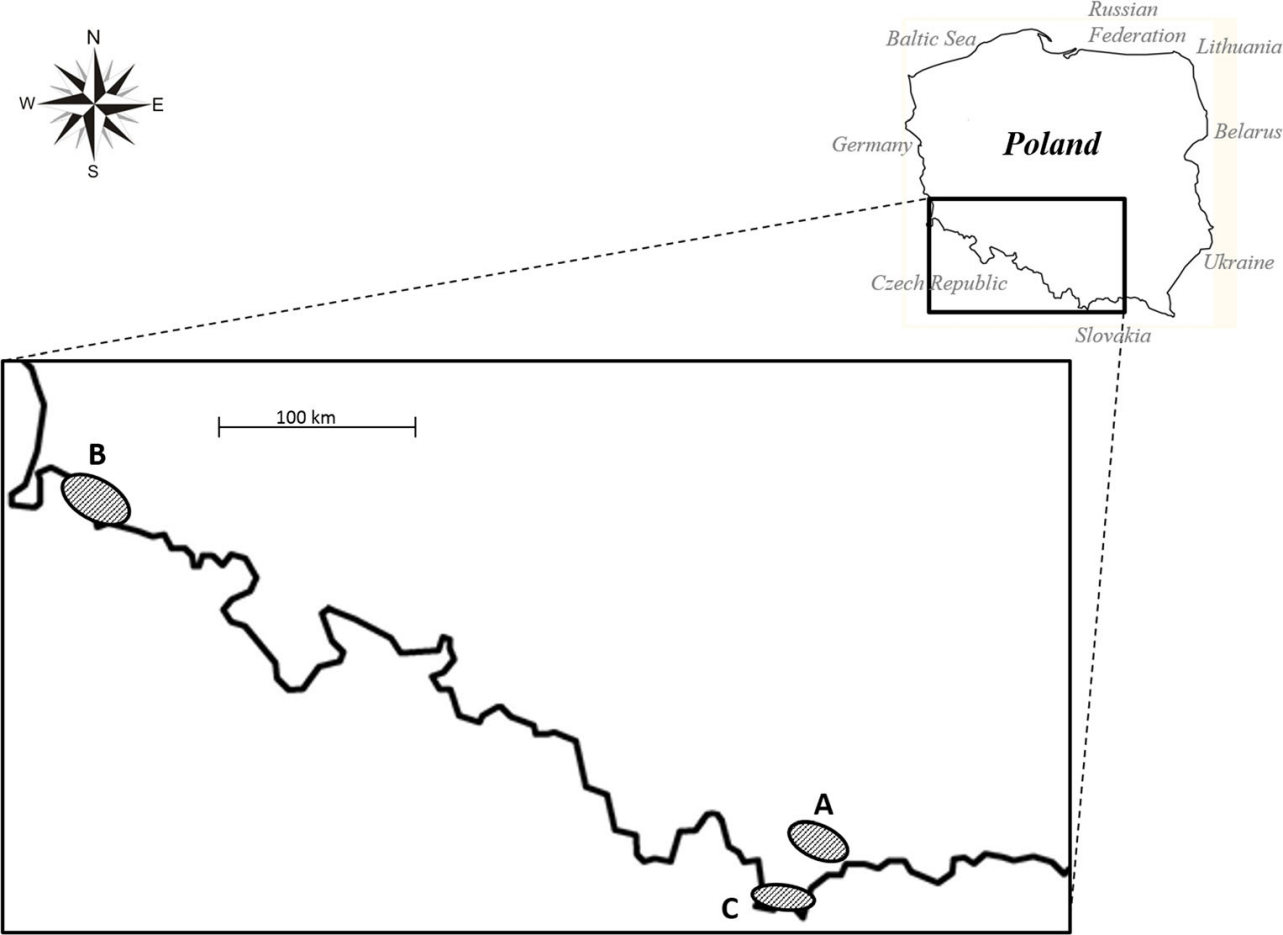
Map displaying the three mountain sampling sites used in this study: A—Gorce Mountains (location A), B—Karkonosze Mountains (location B), C—Tatra Mountains (location C)

The pieces of decaying wood were collected in July 2009, 2010 and 2011 from the floor of forests in the Tatra Mountains (The Tatra National Park), Gorce Mountains (The Gorce National Park) and Karkonosze Mountains (The Karkonosze National Park) located in Central Europe (Fig. 1). The Tatra National Park (49°10′N 19°55′E) and The Gorce National Park (49°35′N 20°3′E) are located in the Carpathian Mountains in Southern Poland. The Tatra National Park is a high mountain national park, ranging from 900 to 2500 m a.s.l., and including vegetation belts from the lower mountain to the alpine zones. The park covers an area of 211.64 km^2^, of which 151.91 km^2^ is forested. The climate is cool and moderately wet. The annual precipitation level is 1800 mm yr^−1^. The average annual temperature is 2–5 °C (Hess 1996; Savva et al. 2006). The Gorce Mts. are relatively low, where the highest peaks exceed 1310 m a.s.l. The area of the Gorce National Park is 70.3 km^2^, of which 65.91 km^2^ is forested. The average annual precipitation varies from 700 mm yr^−1^ in the foothills to 1200 mm yr^−1^ at the highest points (Miczyński 2006). The average annual air temperature varies along the mountains’ longitudinal profile from 6 to 7 °C at the foot of the range to 3 °C along the ridges (Hess 1965). The Karkonosze National Park is located in southwestern Poland, in the Karkonosze Mountains. The park, with an area of 55.76 km^2^, protects the highest part of the Sudeten massif, including Mount Śnieżka, which reaches a height of 1602 m a.s.l. The climate of the Karkonosze is controlled by maritime polar air masses derived from the Atlantic Ocean, which give rise to a local alpine climate. Average total annual precipitation ranges from 1042 mm yr^−1^ at lower elevations to 1102 mm yr^−1^ on Mount Śnieżka. The average annual temperature is 1–7.4 °C (Migała 2005). Karkonosze National Park in Poland and the Krkonoše National Park in the Czech Republic constitute a cross-border biosphere reserve within the framework of the UNESCO Man and the Biosphere Programme (UNESCO 2011).

The pieces of wood with white or brown rot were packed in paper bags, dried at room temperature if wet, and stored until isolation. In the laboratory, small sterile samples (0.5 × 0.2 cm) were taken from each piece of wood, plated on Synthetischer Nährstoffarmer Agar (SNA, Nirenberg 1976) and incubated at 20 °C for 6 days. All colonies resembling *Trichoderma* were twice subcultured on potato dextrose agar (PDA, Oxoid, UK) and incubated as above. Pure cultures were subsequently transferred to tubes containing SNA and stored at 4 °C for further study.

### Identification of Trichoderma

*Trichoderma* isolates obtained in this study were identified at the species level by a combination of morphological and molecular analyses. The morphological characteristics were based on the key by Gams and Bissett (1998) and Chaverri et al. (2015). Colony characteristics were examined in cultures grown on PDA and SNA, after 14 days incubation at a temperature of 25 °C. Microscopic observations were performed in cultures grown on SNA. The molecular identification was based on the sequencing of internally transcribed spacer regions 1 and 2 (ITS1 and ITS2) of the rRNA gene cluster and a fragment of the translation-elongation factor 1-alpha (*tef1*) gene. Mycelium for DNA extraction was obtained as described previously (Błaszczyk et al. 2011). Isolation of total DNA was performed using the CTAB method (Doohan et al. 1998). The ITS1 and ITS2 region of the rDNA gene cluster was amplified using primers ITS4 and ITS5 (White et al. 1990). A fragment of the 1.2-kb *tef1* gene was amplified using primers Ef728M (Carbone and Kohn 1999) and TEF1LLErev (Jaklitsch et al. 2005). The PCR reactions and electrophoresis were carried out under the conditions described by Błaszczyk et al. (2013). The 0.4-kb ITS and 1.2-kb *tef1* amplicon purification steps and sequencing were carried out as described by Błaszczyk et al. (2011). Sequences were edited and assembled using Chromas v. 1.43 (Applied Biosystems). For species identification, sequences were subjected to analysis by BLASTn (http://blast.ncbi.nlm.nih.gov/) as well as *Trich*OKEY and *Tricho*BLAST (http://www.isth.info; Druzhinina et al. 2005; Kopchinskiy et al. 2005; Chaverri et al. 2015). The comparative analyses were based on the ITS and *tef1* sequences of the 119 *Trichoderma* strains determined in this and our previous studies (Jeleń et al. 2014). For comparative analysis, the sequences were aligned using Clustal W program (Thompson et al. 1994). The sequence representing identical allele in the locus of ITS or *tef1* were grouped together. In each group, only one sequence was selected and deposited in the NCBI GenBank (Table 1).

### Phylogenetic analysis

The phylogenetic analyses were based on the selected ITS and *tef1* gene sequences (each allele identified in ITS and *tef1* locus) obtained in this work as well as on the sequences retrieved from NCBI GenBank (http://www.ncbi.nlm.nih.gov) as the closest matches: DQ109573, EU280135, DQ677646, DQ677647, DQ677648, DQ841735, DQ323428, DQ323431, DQ841745, FJ860726, EU871028, FJ860763, FJ442236, KP115288, FJ442276, KP115287, FJ442273, AY737760, EU280074, FJ860799, EU401564, X93963, DQ672607, DQ672606, DQ672605, EU871012, FJ860611, DQ841725, DQ841721, FJ860642, DQ109549, FJ179578, EU401613, AY737736, KP115273, FJ463360, KJ665699, FJ463289, KP115274, FJ463395, JN175595, EU280051. *Fusarium graminearum* (CBS 131784: JX162387, JX118997) was chosen as outgroup. DNA sequences were initially aligned with CLUSTAL W (Thompson et al. 1994) and then rechecked and adjusted manually as necessary using MEGA4 software version (Tamura et al. 2007). Phylogenetic relationships were reconstructed with the MEGA4 software version using the maximum parsimony approach (close-neighbor-interchange algorithm with search level 1 in which the initial trees were obtained with the random addition of sequences—ten replicates) with complete deletion option (i.e. all positions containing gaps and missing data were eliminated from the dataset). All reconstructions were tested by bootstrapping with 1000 replicates.

### Enzyme production

*Trichoderma reseei* QM 9414, sourced from the Czech Collection of Microorganisms (CCM), Brno, Czech Republic was used as the reference strain. The *Trichoderma* collection of 119 isolates were screened for cellulase and xylanase activity under the same conditions, specifically optimized for cellulase/xylanase production with *T. reseei* QM 9414 strain. *Trichoderma* isolates were first cultured on PDA medium in Petri dishes for 3 days at 25 °C. Following incubation, the agar discs (4 mm in length, 3 mm thick) were cut from the sporulated cultures and transferred into 30 mL of autoclaved induction medium (pH 5.5) consisting of (per liter of distilled water) 10 g carboxymethyl cellulose (CMC, Sigma Aldrich, USA), 2 g ammonium phosphate, 2 g potassium orthophosphate, 0.3 g magnesium sulfate, 0.3 g calcium chloride, 1 g bacteriological peptone (Fluka, Switzerland), 2 g yeast extract (Oxoid, UK), 30 mg libremix (CIDA, UK) and 0.001 g cobaltous chloride. The incubation was carried out in 100 ml Erlenmeyer flasks without shaking at 25 °C and optimally for 5 days (in the case of cellulase production) and 7 days (in the case of xylanase production). All analyses were done in triplicate.

### Assay of enzyme activities

After culturing in induction medium, mycelial pellets were removed by centrifugation (15 min at 12,000 *g*). Total cellulase activity was determined using the filter paper activity (FPA) assay (Ghose 1987). Whatman No. 1 filter paper (50 mg, 1 × 6 cm strip; Whatman International, UK) was incubated for 60 min in 0.5 mL of 0.05 M Na-citrate buffer solution (pH 4.8) at 50 °C with an addition of 0.5 mL enzyme solution (supernatant). The reaction mixture was incubated at 50 °C for 15 min. Released reducing sugars were determined by dinitrosalicylic acid reagent (DNS) method of Miller (1959) and Eveleigh et al. (2009), by adding 1 mL of DNS solution and then incubating the mixture at 95 °C for 5 min. Absorbance was measured at 540 nm (UV-1800 Spectrophotometer, Shimadzu, Japan). A calibration curve was established with glucose (POCH, Poland). Final cellulase activity was expressed in filter paper units (FPU) defined as the amount of enzyme which forms 1 μmol of glucose per minute under the assay conditions. Three independent experiments were run for each strain (including blanks).

Xylanase activity was determined by measuring the total amount of reducing sugars released from 1 % (*w/v*) birch wood xylan (Sigma-Aldrich, USA) in 0.5 mL of 50 mM citrate buffer (pH 4.8), supplemented with 0.5 mL enzyme solution (supernatant). The reaction mixture was incubated at 50 °C for 15 min. Released reducing sugars were determined by dinitrosalicylic acid reagent (DNS) method of Miller (1959) and Eveleigh et al. (2009), by adding 1 mL of DNS solution and then incubating the mixture at 95 °C for 5 min. Absorbance was measured at 540 nm (UV-1800 Spectrophotometer, Shimadzu, Japan). A standard curve was prepared with d-xylose (Sigma). The results were expressed in units defined as the amount of enzyme releasing 1 μmol reducing sugar (measured as xylose) during 1 min under the assay conditions. Three independent experiments were run for each strain (including blanks).

### Data analysis

Shannon’s biodiversity index (H) was calculated according to the formula: H = −Σpi * lnpi, where pi is the proportion of ith species (Shannon 1948). The Shannon evenness (E) was calculated according to the equation: E = H/lnS, where S is the total number of species. The Simpson’s biodiversity index (D) was calculated as D = 1 − Σi ni (ni − 1)i / N(N − 1), where ni represents specimens of a species and N represents the total number of specimens (Simpson 1949).

The statistical analysis was performed using the programs Statistica version 10.0 (StatSoft Inc.) and Microsoft Excel version 2010. Inference about the significance of differences between enzyme activity of isolates was conducted using one-way ANOVA and was determined using Tukey’s test (*α* = 0.05).

## Results

### Identification of *Trichoderma*

One hundred and four *Trichoderma* isolates obtained from samples of decaying wood collected in the forests of the Tatra Mountains (location C), Gorce Mountains (location A) and Karkonosze Mountains (location B) in Poland were identified at the species level based on morphological as well as ITS1, ITS2 and *tef1* sequencing data. Finally, 12 species or species complex were found: *T. atroviride*, *T. citrinoviride*, *T. cremeum* P. Chaverri & Samuels, *T. gamsii*, *T. harzianum* complex, *T. koningii*, *T. koningiopsis*, *T. longibrachiatum*, *T. longipile* Bissett, *Trichoderma* sp. (*H. parapilulifera* B.S. Lu, Druzhin. & Samuels)*, T. viride* Schumach. and *T. viridescens* complex. Furthermore, the *tef1* sequences analysis of the isolates belonging to the *T. harzianum* species complex and *T. viridescens* species complex revealed five isolates as *Trichoderma atrobrunneum* F.B. Rocha, P. Chaverri & W. Jaklitsch, 11 isolates as *Trichoderma harzianum* Rifai, one isolate as *Trichoderma viridarium* Jaklitsch, Samuels & Voglmayr, three isolates as *Trichoderma trixiae* Samuels & Jaklitsch and ten isolates as *Trichoderma paraviridescens* Jaklitsch, Samuels & Voglmayr. The identification, origin and NCBI GenBank accession numbers of all isolates are given in Table 1. In order to determine the relationship among *Trichoderma* strains originated from decayed wood collected in a mountain forest in Poland and selected from NCBI GenBank as close relatives, the phylogenetic analysis were performed. The results were presented as a consensus ITS tree and *tef1* tree (Online resource 1, 2).

### Species diversity and distribution

The analysis were based on the 119 *Trichoderma* strains occurring on wood with decay symptoms in mountain forests in Central Europe, identified in this study and published previously by Jeleń et al. (2014). Grouping of the species according to their geographic origin, the same number (8) of species was found in location A and C, with a slightly lower number (5) in location B (Table 1). However, the proportion of isolates obtained from location C was the highest (69 isolates, 58 %), followed by that recovered from location B (26 isolates, 22 %) and location A (24 isolates, 20 %). Some of the species were unique to a particular location—*T. cremeum*, *T. longibrachiatum* and *T. koningii* were only collected from location A, *T. koningiopsis* only from location B and *T. gamsii*, *T. longipile* and *Trichoderma* sp. (*H. parapilulifera*) only from location C. On the other hand, four species (*T. atroviride*, *T. citrinoviride*, *T. viride* and *T. viridescens* complex) were found in all the locations. Among the 12 species described in this study, *T. viride* was the most abundant species (53 % of all isolates). It was also observed as the most dominant fungus in all the locations investigated. Another very common species complex (17 % of all isolates) occurring in the three locations was *T. viridescens*. After *T. viride* and *T. viridescens* complex, *T. harzianum* was commonly isolated (13 % of all isolates) from samples of decaying wood. However, the presence of this species complex was mainly ascertained in location C. Whereas, the other species (*T. atroviride, T. citrinoviride, T. cremeum, T. gamsii, T. koningii, T. koningiopsis, T. longibrachiatum, T. longipile* and *H. parapilulifera*) were represented by several strains and accounted for the minority of isolates (17 % of all isolates).

The obtained data were used to calculate the Shannon’s biodiversity index (H), evenness (E) and the Simpson’s biodiversity index (D) for each location and the total population. The highest species diversity and evenness were recorded for location A (H′ = 1.71, *E* = 0.82, *D* = 0.79). The Shannon and Simpson diversity indexes, estimated for the other two sites showed, that location C had higher species diversity (H′ = 1.34, *D* = 0.64) than location B (H′ = 1.08, *D* = 0.58), while the species evenness measure resulted in *E* = 0.67 for location B and *E* = 0.64 for location C. Consequently, location C was characterized by the least homogenous distribution of species. The overall diversity values for *Trichoderma* species originating from decaying wood in Polish mountains were H′ = 1.53, *E* = 0.62 and *D* = 0.67.

### Cellulolytic and xylanolytic activities

**Fig. 2:**
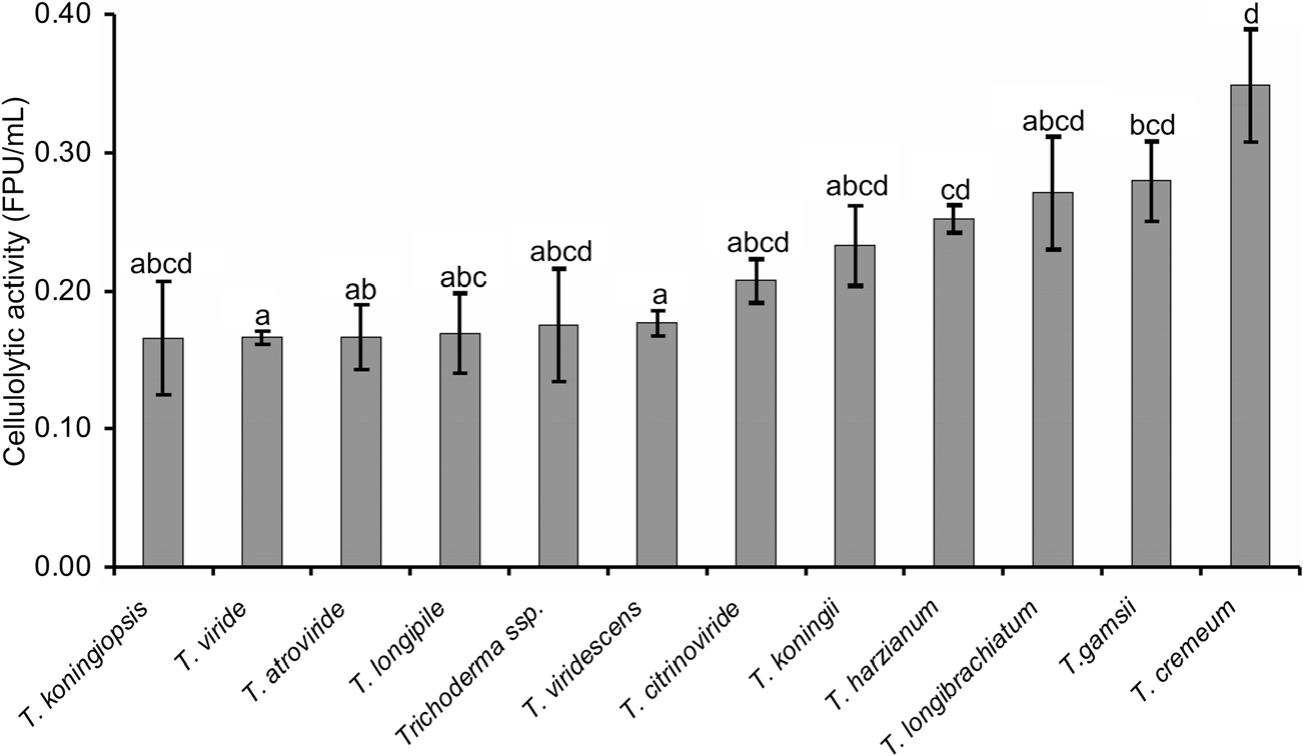
Total cellulase activity distribution for eight *Trichoderma* species. Different lowercase letters indicate significant difference in enzymatic activity among species. *Bars* represent mean ± standard error. *Trichoderma harzianum* include isolates *T. harzianum* and *T. atrobrunneum*, belonging to the *T. harzianum* species complex. *Trichoderma viridescens* include isolates *T. paraviridescens*, *T. trixiae* and *T. viridarium*, belonging to the *T. viridescens* species complex. *Bars* represent mean ± standard error of three independent analyses (assay of cellulase activity) for each strain

**Fig. 3:**
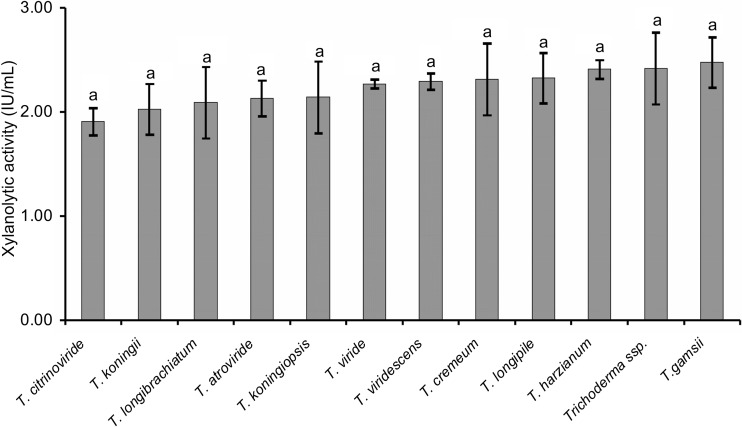
Xylanase activity distribution for eight *Trichoderma* species. The same lower case letters indicate non-significant differences in xylanolitic activity among species. *Trichoderma harzianum* include isolates *T. harzianum* and *T. atrobrunneum*, belonging to the *T. harzianum* species complex. *Trichoderma viridescens* include isolates *T. paraviridescens*, *T. trixiae* and *T. viridarium*, belonging to the *T. viridescens* species complex. *Bars* represent mean ± standard error of three independent analyses (assay of xylanase activity) for each strain

A total of 119 *Trichoderma* isolates—104 isolates identified in the present paper and 15 isolates described previously by Jeleń et al. (2014), belonging to 12 species or species complex and originating from decaying wood collected in Polish mountains, were examined for their ability to produce cellulolytic and xylanolytic enzymes. All of the isolates were able to degrade cellulose (Whatman No. 1 filter paper) and birch wood xylan (Online resource 3). However, the levels of cellulase and xylanase activities were differential both between isolates of the same species (in the case of species represented by at least two isolates, such as *T. atroviride*, *T. gamsii*, *T. harzianum* complex, *T. koningii*, *T. viride*, *T. viridescens* complex) and between species (Figs. 2 and 3). The highest total cellulase activity (FPA) was observed for *T. cremeum* species, represented by only a single isolate, AN392 (Fig. 2). Also, *T. gamsii*, especially isolate AN327 (0.308 ± 0.006 FPU/mL), and *T. longibrachiatum* species were found to be efficient producers of cellulolytic activity. *Trichoderma harzianum* complex*, T. koningii* and *T. citrinoviride* species displayed moderate cellullase activity. A lower cellulose-decomposing potential was recorded for *T. koningiopsis, T viride, T. atroviride*, *T. longipile*, *Trichoderma* sp. (*H. parapilulifera*) and *T. viridescens* complex. Considering the diversity within *T. atroviride* species, cellulase activity ranged from 0.135 ± 0.001 (isolate AN705) to 0.201 ± 0.035 (isolate AN240) FPU/mL and within *T. citrinoviride* species, this activity ranged from 0.135 ± 0.002 (isolate AN710) to 0.281 ± 0.009 (isolate AN303b) FPU/mL. The activity produced by two isolates of *T. koningii* (AN398 and AN399) was 0.267 ± 0.001 and 0.200 ± 0.028 FPU/mL, respectively. In the set of 16 isolates of *T. harzianum* complex there were isolates (AN370, AN704, AN364, AN706, AN699) identified as *T. atrobrunneum*, that showed weak cellulose-decomposing potential, but there were also five isolates of *T. harzianum* (AN415, AN394, AN373, AN367, AN312) with high decomposition activity towards cellulose (over 0.3 FPU/mL). Cellulase activity displayed by isolates belonging to *T. viridescens* species complex ranged from 0.074 ± 0.003 (*T. paraviridescens* AN328) to 0.271 ± 0.002 (*T. paraviridescens* AN702) FPU/mL. However, the variation in cellulase activity differed significantly among the largest group of *T. viride* isolates, the lowest value being 0.075 ± 0.004 (isolate AN340), whereas the highest was as high as 0.283 ± 0.000 FPU/mL for isolate AN701.

As shown in Fig. 3, non-significant differences in the xylanase activity were obtained between species. Of all species, *T. gamsii* was found to be the best producer of xylanolytic activity (over 2.4 U/mL), in contrast to *T. citrinoviride*, which was characterized as the least effective decomposer of xylan (Fig. 3, Online resource 3).

However, the variation in xylanolytic activity differed significantly among the *T. citrinoviride* isolates, the lowest value being 1.147 ± 0.003 U/mL (AN710), whereas the highest was 2.396 ± 0.011 U/mL (AN499). Similarly, xylanolytic activity varied among isolates of *T. atroviride* from 1.808 ± 0.003 (AN 705) to 2.519 ± 0.006 (AN497) U/mL. Within *T. harzianum* species complex, the highest xylanase activity was observed for the *T. harzianum* AN369 isolate (2.887 ± 0.022 U/mL) and the lowest for the *T. harzianum* AN480 isolate (1.634 ± 0.001 U/mL). Xylanase activities of two *T. koningii* isolates, AN399 and AN398, were 2.001 ± 0.0077 and 2.052 ± 0.095 U/mL, respectively. Among the 63 isolates of *T. viride*, the most effective in xylan degradation were three isolates: AN813 (3.100 ± 0.016 U/mL), AN810 (3.076 ± 0.008 U/mL) and AN827 (3.070 ± 0.008 U/mL), whereas AN242 was observed to be a weak decomposer of cellulose (1.227 ± 0.004 U/mL). In the set of 20 isolates of *T. viridescens* species complex, the highest levels of xylanase activity were achieved by the *T. paraviridescens* AN702 (3.099 ± 0.004 U/mL) and AN609 (3.070 ± 0.005 U/mL) and the lowest was presented by the *T. paraviridescens* AN709 isolate (0.946 ± 0.001 U/mL).

## Discussion

The present study yielded a total of 12 species of *Trichoderma*. Eight *Trichoderma* species and species complex, namely *T. atroviride, T. citrinoviride, T. harzianum* complex*, T. koningii, T. viride, T. viridescens* complex*, T. koningiopsis* and *T. gamsii*, have also been isolated from decaying wood collected in parks and forests of a lowland region of Poland in our preliminary investigations (Błaszczyk et al. 2011). It is worth noting that in contrast to the previous study, where *T. harzianum* was the most frequently isolated species complex from decaying wood, here *T. viride* was observed to be the predominant taxon associated with this microhabitat. The numerous reports on *T. viride* and its close relative *T. viridescens* species complex in the literature imply a wide geographic distribution for these species, but Samuels (2006), Jaklitsch et al. (2006b) and Jaklitsch (2011) observed it to be more common in north- and south-temperate regions, including Europe (Austria, Czech Republic, England, France, Germany, Northern Ireland, Russia, Sweden), Japan, New Zealand, Canada and the USA. These species have been isolated from various materials, especially from soil, peat, wood and leaf litter. In Europe, the stroma of its teleomorph have been found inconspicuously on wood and bark of cut branches, tree tops or logs of deciduous and coniferous trees (Jaklitsch 2011; Jaklitsch et al. 2013). A recent study of Jaklitsch et al. (2013) provided details of the *T. viridescens* species complex, including the species identified in the present study, namely *T. paraviridescens, T. trixiae* and *T. viridarium*. Jaklitsch et al. (2013) contend that all these species are known to occur in Europe, although the commonest species of the *T. viridescens* complex in Europe is *T. paraviridescens. Trichoderma viridarium* is particularly common in southern Europe. Whereas *T. trixiae* and exactly its sexual morph has been found only once in Germany in immature condition. As shown here, after *T. viride* and *T. viridescens* species complex, *T. harzianum* complex was commonly isolated (13 % of all isolates) from samples of decaying wood. Recently, the taxonomic of *T. harzianum* species complex was revised to include at least 14 species (Chaverri et al. 2015). Two of these species, namely *T. harzianum* and *T. atrobrunneum*, have been identified in the present study. As reported by Chaverri et al. (2015), *T. harzianum* is an uncommon species known only from Europe and North America, isolated mostly from soil and mushroom compost and occasionally as an endophyte in stems. According to these authors, the geographic distribution of *T. atrobrunneum* is also restricted to temperate regions (Europe, North America). However, this species has been isolated from soil, decaying wood and other fungi (Chaverri et al. 2015). The other species recognized in the present study, such as *T. atroviride*, *T. citrinoviride, T. longibrachiatum*, *T. gamsii* and *T. koningiopsis*, are known from their evident widespread occurrence in Europe, North and South America and Asia (Belayneh Mulaw et al. 2010; Hoyos-Carvajal et al. 2009; Jaklitsch 2009, 2011; Kubicek et al. 2003; López-Quintero et al. 2013; Zhang et al. 2005), whereas *T. longibrachatum* and *T. koningiopsis* are preferentially isolated from warmer or tropical areas. However, *T. koningii* and *T. longipile* exhibit a distribution restricted to Europe and North America (Kubicek et al. 2008). The latter has so far only been isolated from wood of *Populus*, wood of *Ulmus* and soil in Europe and Canada (Chaverri et al. 2003; Jaklitsch 2011). A rare species, whose supposed anamorph—*Trichoderma* isolate AN471—was found in the present study, is *H. parapilulifera*. This species is mainly known from two teleomorphic specimens collected in North America and Europe, and its anomarphic state was detected and characterized recently by Jaklitsch (2011). However, there has been little study to date of the occurrence of *T. cremeum* species, exclusively (isolate AN392) obtained in the present research from decaying wood in the Gorce Mountains (location A). Previously, only its teleomorph (*Hypocrea cremea*) had been found by Chaverri et al. (2003) in North America and New Zealand.

Our study showed the highest species diversity and evenness at location A (Gorce Mountains), and the lowest levels of species richness at location B (Karkonosze Mountains). We attribute this phenomenon to differences in the biodiversity of trees, and consequently, the type of wood at the different locations. The majority of the Karkonosze National Park consists of forests dominated by *Picea abies* (L.) H. Karst. (Hess 1965). The vegetation of the Gorce Mts is composed chiefly of deciduous and coniferous forests, in a great part primeval (Carpathian Primeval Forest) and natural (Loch and Armatys 2008). The most common species of trees are *Picea abies* (L.) H. Karst, *Fagus sylvatica* L., and *Abies alba* Mill., while admixture species include larch, elm, ash and grey alder (Loch and Armatys 2008). Whereas, the forests of the Tatra Mountains are dominated by *Picea abies* (L.) H. Karst with low occurrence *Abies alba* Mill. and *Fagus sylvatica* L. and isolated individuals of *Pinus cembra* L. and *Larix decidua* Mill. (Fleischer et al. 2009). Both the Gorce National Park and the Tatra National Park were characterized by a similar diversity of *Trichoderma* species. It is worth noting that the distribution of these species at the Tatra National Park (location C) was found as the least homogenous. It is worth recalling that in November 2004, spruce stands in the Tatra National Park on the area of 12,000 hectares were affected by a catastrophic windthrow and 1 year later (in July 2005) wildfire broke out on a part (220 ha) of this area (Fleischer et al. 2009; Kopecká and Nováèek 2009). This may have contributed to the decline of the originally present abundance of species dependent on forest, their diversity and redistribution in Tatra National Park (Fleischer et al. 2009; Kopecká and Nováèek 2009).

All of the 119 *Trichoderma* strains investigated in this study were isolated from pieces of wood with decay symptoms. Consequently, the question arose as to whether all these strains are able to participate in decomposition of particularly xylan and cellulose containing organic matter. The cellulolytic and xylanolytic activity assay is one of the approaches to the preliminary determination of the potential of the strains for enzymes production and has been widely used in many studies for screening of a broad spectrum of fungal isolates and environmental samples (Benoliel et al. 2013; Cianchetta et al. 2010; Delabona et al. 2012; Ja’afaru 2013). This preliminary screening showed that all *Trichoderma* species identified in the present study disclose the potential to degrade cellulose (Whatman No. 1 filter paper) and birch wood xylan under assay conditions used. The possibility of cellulase and hemicellulase complex formation by *T. atroviride*, *T. citrinoviride*, *T. gamsii*, *T. harzianum*, *T. koningii*, *T. longibrachiatum*, *T. viride* and *T. viridescens* complex, has already been stated in previous studies (Gusakov 2011; Strakowska et al. 2014; Targoński 1991). To the best of our knowledge, this is the first study revealing the ability of *T. cremeum*, *T. longipile* and *H. parapilulifera* towards cellulase and xylanase production. However, the results of this work revealed the same variability in the level of enzymatic activity among *Trichoderma* species and even among strains of the same species. It will be interesting to study further what causes these differences in hydrolytic activity among the isolates. The explanation of this question seems to be important due to the potential use of the tested strains for further biotechnological purposes.

The present study has shown the real potential of all *Trichoderma* species originating from wood with decay symptoms to produce cellulases and xylanases—the key enzymes in plant cell wall degradation, and thus to a saprotrophic style of nutrition. Interestingly, some of these species are known to be both saprotrophic and mycoparasitic or additionally exist as endophytes (*T. gamsii*, *T. harzianum*) and immuno-compromised human pathogens, such as *T. longibrachiatum* (Druzhinina et al. 2011; Jaklitsch 2009) or *T. gamsii* (Rinu et al. 2014). However, there is still no definitive statement as to which of these species living on decaying wood is a strong saprotroph or strong mycoparasite or which species uses both ways of nutrition and what determines the switching between these two tropic strategies. The ability of *Trichoderma* to derive nutrients from other fungi mediated by mycoparasitism has been clearly investigated in species of *T. harzianum*, *T. atroviride* and *T. viride* (Druzhinina et al. 2011). Based on recent evolutionary studies, the mycoparasitic way of nutrition has also been deduced for *T. cremeum*, *T. longipile*, *T. viridescens*, *T. koningii* and *T. koningiopsis*, while *T. citrinoviride, T. longibrachiatum* and *H. parapilulifera* species are predicted to function as saprotrophs. According to Chaverri and Samuels (2013), the association of *Trichoderma* species with plants (saprotrophy, plant necrotrophy) has evolved at least twice (from mycoparasitism to saprotrophy: *T. citrinoviride*, *T. longibrachiatum*, *H. parapilulifera*) and then reversed to mycoparasitism possibly twice (from mycoparasitism to mycoparasitism by saprotrophism: *T. atroviride*, *T. gamsii*, *T. viride*, *T. viridescens*, *T. koningii*, *T. koningiopsis*). Chaverri and Samuels (2013) also observed that the habitat preference (decaying plants, soil, fungi, living plants) of *Trichoderma* has been gained or lost multiple times in the evolution of this genus and the majority of these changes have concerned the shift from decaying plant material to soil or fungi, from soil to endophyte and from fungi to decaying plant material. Xie et al. (2014) concluded that the development of a new way of nutrition is not only related to the decrease of nutrition-related genes (as in the case of mycoparasitism-specific genes), but also to the increase of selection pressure on nutrition-related genes (as in the case of genes encoding the plant cell wall degrading enzymes).

Considering our findings with respect to genomic and transcriptomic studies and evolutionary investigations, it is only possible to infer the capacity of a single *Trichoderma* species to use any of these ecological strategies. However, further studies are needed to identify the key elements in the molecular repertoire required for balancing between the saprotrophic and mycoparasitic ways of nutrition of putative *Trichoderma* species.

## Electronic supplementary material

Below is the link to the electronic supplementary material.

Online resource 1 The consensus tree of maximum parsimony analyses based on ITS sequences dataset of 19 selected *Trichoderma* strains investigated in the present study (the sequences representative of each allele identified in ITS locus) and 22 sequences retrieved from GenBank. *Fusarium graminearum* was used as an outgroup in the analysis. The ITS sequences analysis involved 42 nucleotide sequences corresponding to a total of 444 characters in the final dataset, of which 318 were conserved, 126 were variable and 86 parsimony informative. Maximum parsimony analysis led to 434 equally parsimonious trees, with similar clade topologies and tree length 107, consistency index CI = 0.850, retention index RI = 0.962 and rescaled consistency index RCI = 0.818. The sequence accession numbers obtained from NCBI GenBank (http://www.ncbi.nlm.nih.gov) are shown in parentheses. Bootstrap values higher than 50 % (1000 replicates) are shown at each branch. (GIF 83 kb)

High resolution (TIF 36716 kb)

Online resource 2 The consensus tree of maximum parsimony analyses based on *tef1* sequences dataset of 19 selected *Trichoderma* strains investigated in present study (the sequences representative of each allele identified in ITS locus) and 22 sequences retrieved from GenBank. *Fusarium graminearum* was used as an outgroup in the analysis. The *tef1* sequences analysis involved 42 nucleotide sequences and there were a total of 463 positions in the final dataset, of which 178 were conserved, 284 were variable and 266 parsimony informative. Maximum parsimony analysis generated 26 equally parsimonious trees of 436 steps length with consistency index CI = 0.566, retention index RI = 0.853 and rescaled consistency index RCI = 0.484. The sequence accession numbers obtained from NCBI GenBank (http://www.ncbi.nlm.nih.gov) are shown in parentheses. Bootstrap values higher than 50 % (1000 replicates) are shown at each branch. (GIF 88 kb)

High resolution (TIF 36764 kb)

Online resource 3 Cellulolytic and xylanolytic activity of 120 isolates belonging to different *Trichoderma* species. Values represent mean ± standard error of three independent analyses (assay of cellulase/xylanase activity) for each strain. (DOC 126 kb)
