# Supplementary figures and images for: Trichoderma species occurring on wood with decay symptoms in mountain forests in Central Europe: genetic and enzymatic characterization
Source: J Appl Genet. 2015 Nov 19;57:397–407. doi: 10.1007/s13353-015-0326-1 (PMC4963455; doi:10.1007/s13353-015-0326-1)

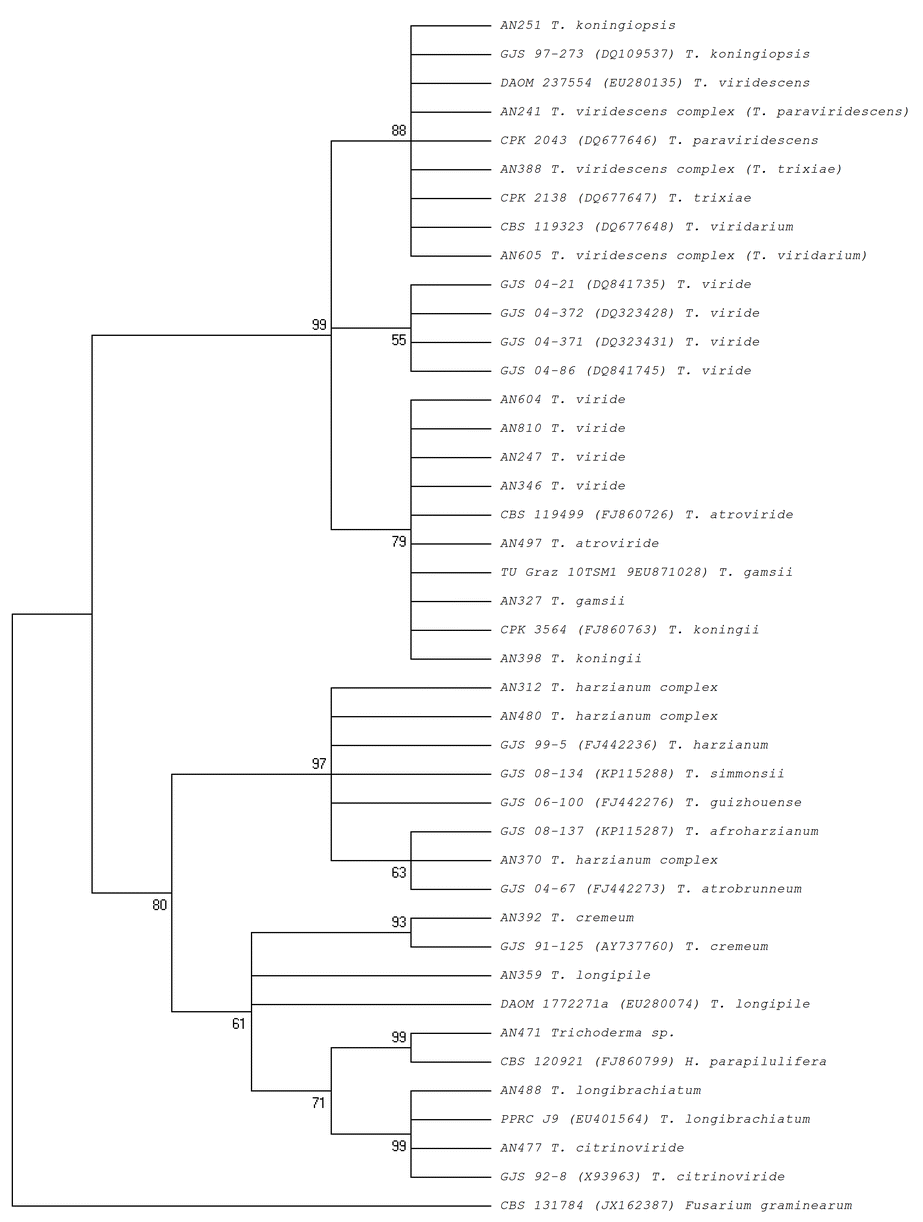

Supplement: Supplementary file 1 — The consensus tree of maximum parsimony analyses based on ITS sequences dataset of 19 selected Trichoderma strains investigated in the present study (the sequences representative of each allele identified in ITS locus) and 22 sequences retrieved from GenBank. Fusarium graminearum was used as an outgroup in the analysis. The ITS sequences analysis involved 42 nucleotide sequences corresponding to a total of 444 characters in the final dataset, of which 318 were conserved, 126 were variable and 86 parsimony informative. Maximum parsimony analysis led to 434 equally parsimonious trees, with similar clade topologies and tree length 107, consistency index CI = 0.850, retention index RI = 0.962 and rescaled consistency index RCI = 0.818. The sequence accession numbers obtained from NCBI GenBank (http://www.ncbi.nlm.nih.gov) are shown in parentheses. Bootstrap values higher than 50 % (1000 replicates) are shown at each branch. (GIF 83 kb) [file 13353_2015_326_Fig4_ESM.gif]

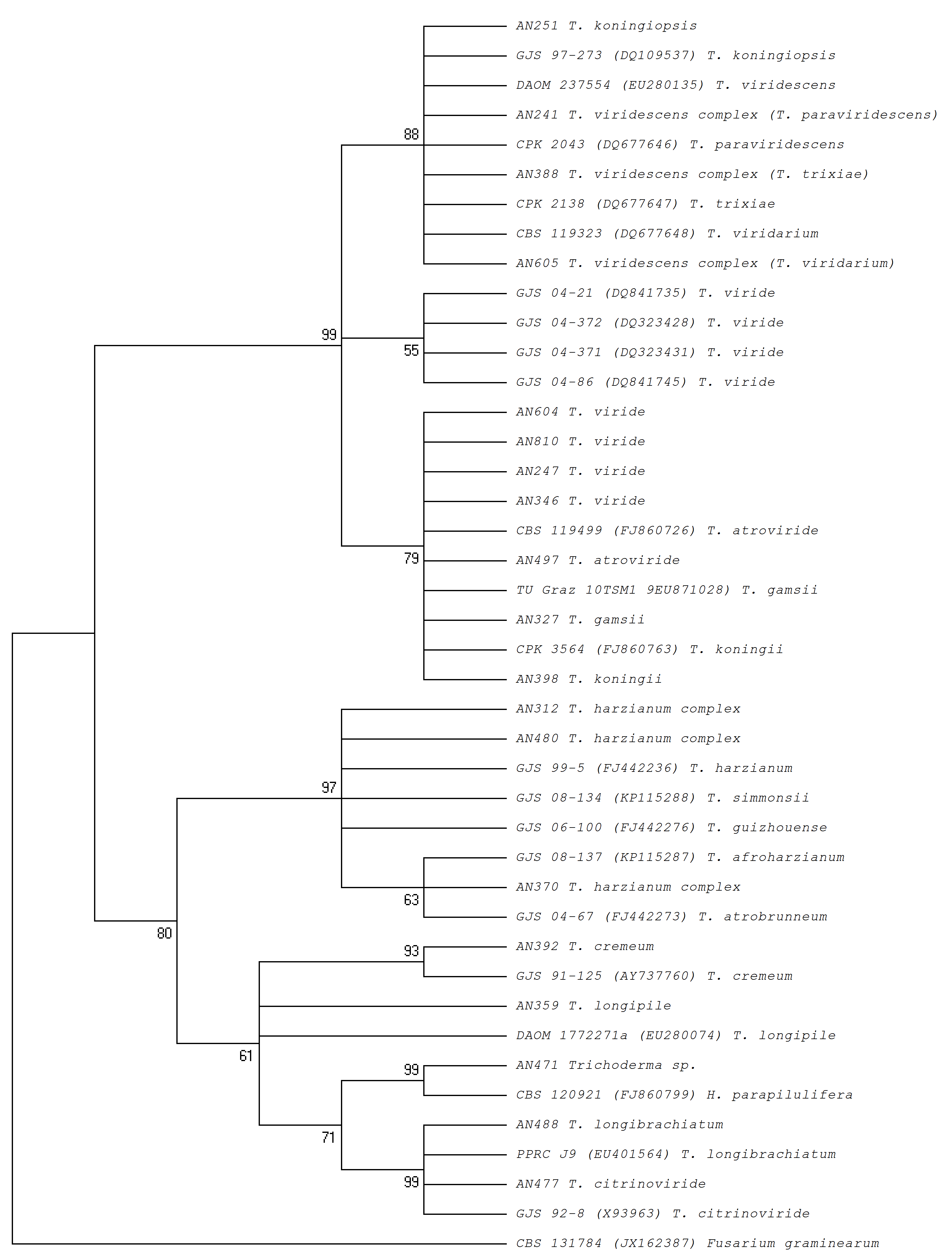

Supplement: Supplementary file 2 — High resolution (TIF 36716 kb) [file 13353_2015_326_MOESM1_ESM.tif]

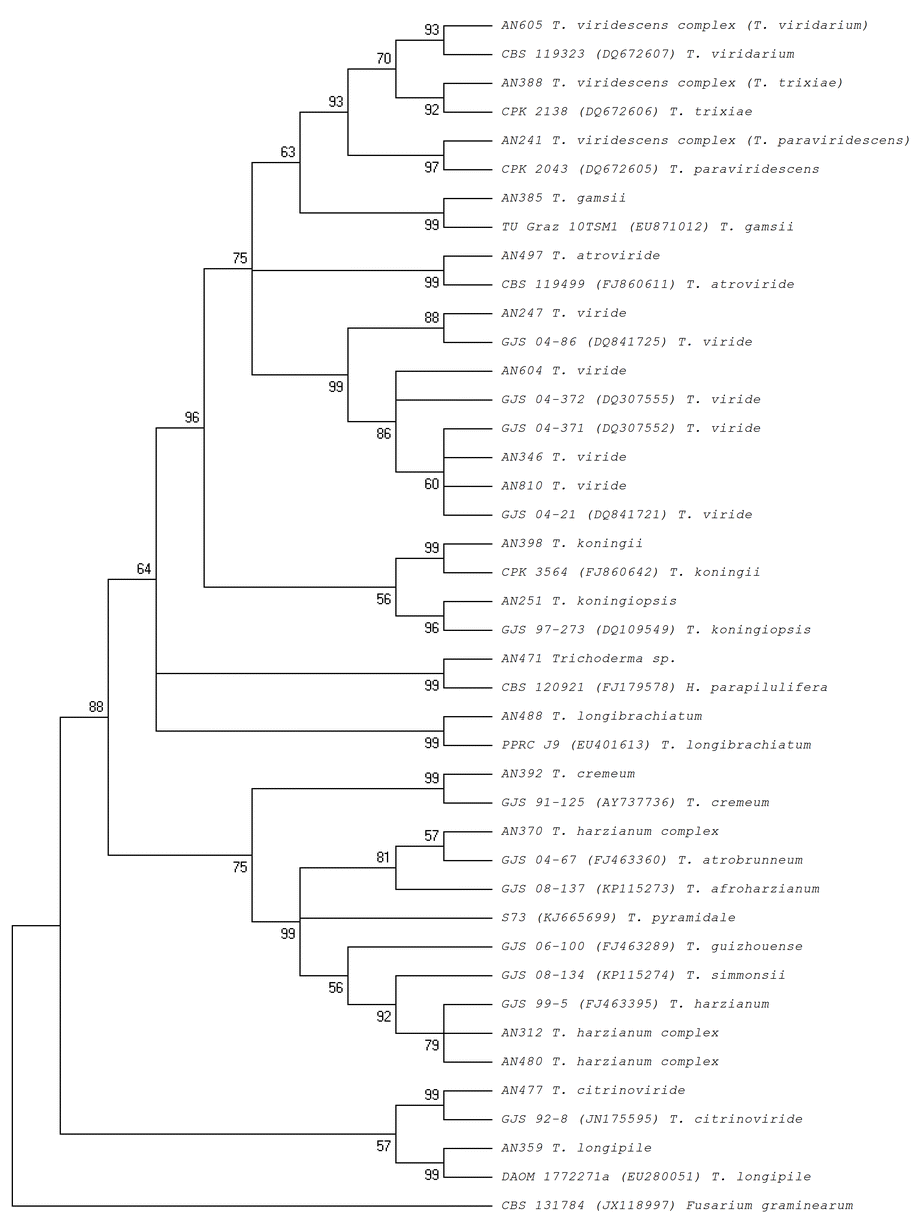

Supplement: Supplementary file 3 — The consensus tree of maximum parsimony analyses based on tef1 sequences dataset of 19 selected Trichoderma strains investigated in present study (the sequences representative of each allele identified in ITS locus) and 22 sequences retrieved from GenBank. Fusarium graminearum was used as an outgroup in the analysis. The tef1 sequences analysis involved 42 nucleotide sequences and there were a total of 463 positions in the final dataset, of which 178 were conserved, 284 were variable and 266 parsimony informative. Maximum parsimony analysis generated 26 equally parsimonious trees of 436 steps length with consistency index CI = 0.566, retention index RI = 0.853 and rescaled consistency index RCI = 0.484. The sequence accession numbers obtained from NCBI GenBank (http://www.ncbi.nlm.nih.gov) are shown in parentheses. Bootstrap values higher than 50 % (1000 replicates) are shown at each branch. (GIF 88 kb) [file 13353_2015_326_Fig5_ESM.gif]

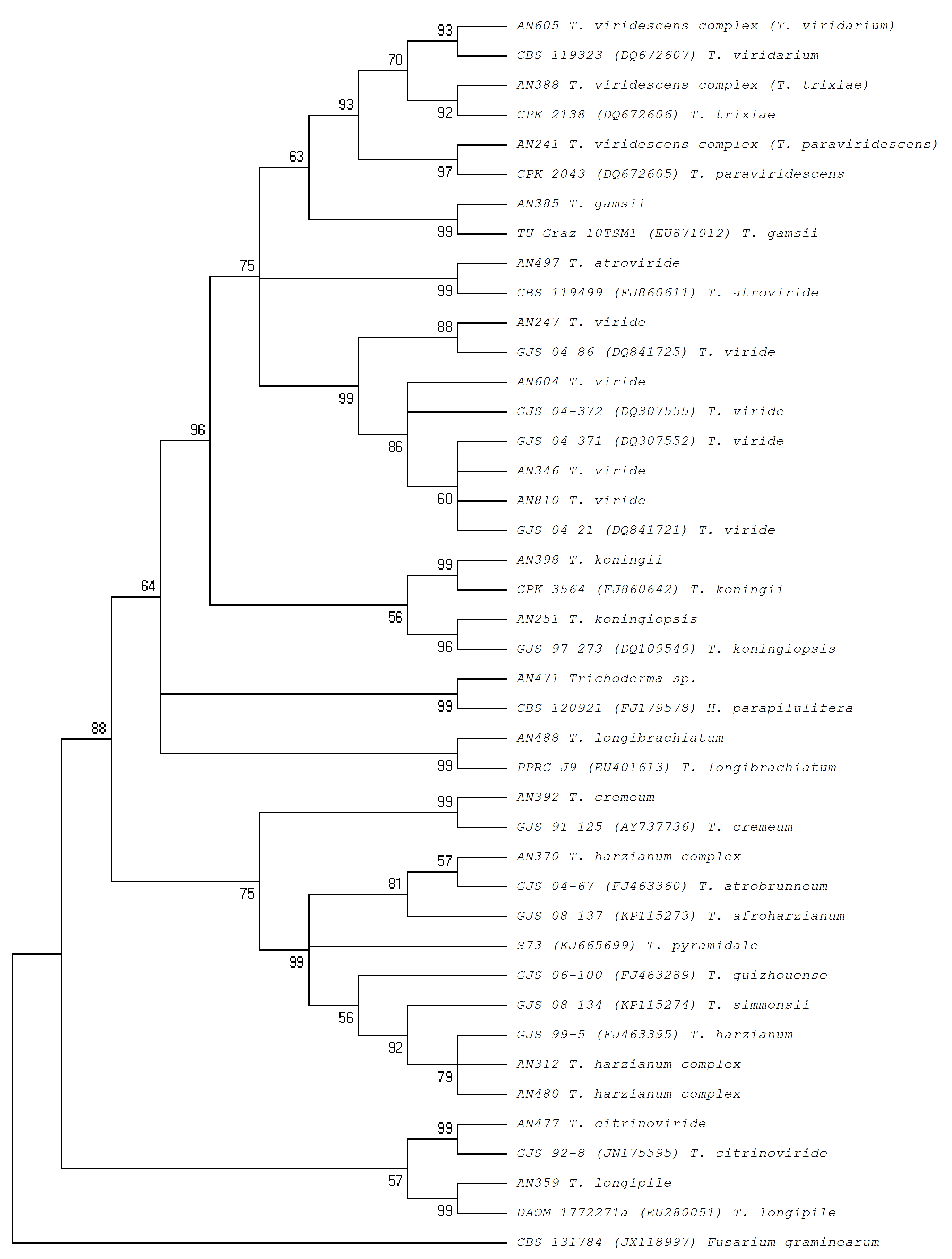

Supplement: Supplementary file 4 — High resolution (TIF 36764 kb) [file 13353_2015_326_MOESM2_ESM.tif]
