# Supplementary material for: Trichoderma species occurring on wood with decay symptoms in mountain forests in Central Europe: genetic and enzymatic characterization
Source: J Appl Genet. 2015 Nov 19;57:397–407. doi: 10.1007/s13353-015-0326-1 (PMC4963455; doi:10.1007/s13353-015-0326-1)
Supplement: Supplementary file 5 — Cellulolytic and xylanolytic activity of 120 isolates belonging to different Trichoderma species. Values represent mean ± standard error of three independent analyses (assay of cellulase/xylanase activity) for each strain. (DOC 126 kb) [file 13353_2015_326_MOESM3_ESM.doc]

Table S1. Cellulolytic (FPU/mL) and xylanolytic (U/mL) activity of tested isolates belonging to different *Trichoderma* species (means ± standard error). The same lowercase letter in a column indicate non - significant differences among isolates

| Isolates | Cellulolytic activity (FPU/mL) | Xylanolitic activity (U/mL) |
| --- | --- | --- |
| ***T. citrinoviride*** |  |  |
| AN710 | 0.135 ± 0.002a | 1.147 ± 0.003 a |
| AN499 | 0.171 ± 0.013 ab | 1.651± 0.053 b |
| AN490 | 0.183 ± 0.024 ab | 1.689 ± 0.132 b |
| AN393 | 0.195 ± 0.069 ab | 1.852 ± 0.010 b |
| AN500 | 0.229 ± 0.016 ab | 2.226 ± 0.017 c |
| AN477 | 0.256 ± 0.034 ab | 2.383 ± 0.020 c |
| AN303b | 0.281 ± 0.009 b | 2.396 ± 0.011 c |
| ***T. harzianum* complex** |  |  |
| AN370 | 0.168 ± 0.027 ab | 1.634 ± 0.001 a |
| AN704 | 0.178 ± 0.032 b | 1.640 ± 0.003 a |
| AN364 | 0.193 ± 0.012 abc | 1.648 ± 0.005 a |
| AN706 | 0.206 ± 0.002 abcd | 2.067 ± 0.181 ab |
| AN699 | 0.207 ± 0.002 abcd | 2.206 ± 0.023 bc |
| AN369 | 0.213 ± 0.012 abcd | 2.453 ± 0.196 bcd |
| AN480 | 0.225 ± 0.000 abcd | 2.466 ± 0.104 bcd |
| AN360 | 0.264 ± 0.026 abcd | 2.488 ± 0.017 bcd |
| AN349 | 0.271 ± 0.006 abcd | 2.552 ± 0.130 bcd |
| AN479 | 0.274 ± 0.037 abcd | 2.671 ± 0.024 cd |
| AN381 | 0.283 ± 0.026 abcd | 2.807 ± 0.040 d |
| AN312 | 0.309 ± 0.007 bcd | 2.838 ± 0.004 d |
| AN367 | 0.312 ± 0.024 bcd | 2.874 ± 0.079 d |
| AN373 | 0.321 ± 0.035 cd | 2.883 ± 0.083 d |
| AN394 | 0.342 ± 0.031 d | 2.887 ± 0.022 d |
| AN415 | 0.346 ± 0.000 d | 1.634 ± 0.001 a |
| ***T. longipile*** |  |  |
| AN359 | 0.177 ± 0.025 a | 2.037 ± 0.008 a |
| AN414 | 0.161 ± 0.015 a | 2.613 ± 0.064 b |
| ***T. cremeum*** |  |  |
| AN392 | 0.348 ± 0.037 | 2.313 ± 0.164 |
| ***T. longibrachiatum*** |  |  |
| AN488 | 0.271± 0.0004 | 2.090 ± 0.1860 |
| ***T. atroviride*** |  |  |
| AN240 | 0.201± 0.035 a | 1.808 ± 0.003 a |
| AN497 | 0.164 ± 0.006 a | 1.905 ± 0.005 b |
| AN705 | 0.135 ± 0.001 a | 2.288 ± 0.001 c |
| ***T. viride*** |  |  |
| AN340 | 0.075 ± 0.004 ab | 1.227 ± 0.004 a |
| AN352 | 0.077 ± 0.049 abc | 1.327 ± 0.001 a |
| AN826 | 0.085 ± 0.033 a | 1.595 ± 0.129 b |
| AN604 | 0.093 ± 0.001 abcd | 1.613 ± 0.039 bc |
| AN374 | 0.093 ± 0.038 abcd | 1.637 ± 0.141 bcd |
| AN687 | 0.096 ± 0.001 abcdf | 1.642 ± 0.019 bcd |
| AN814 | 0.097 ± 0.001 abcdf | 1.657 ± 0.002 bcd |
| AN802 | 0.097 ± 0.001 abcdf | 1.660 ± 0.001 bcd |
| AN347 | 0.107 ± 0.023 abcdfg | 1.679 ± 0.029 bcd |
| AN827 | 0.109 ±0.001 abcdfg | 1.692 ± 0.023 bcd |
| AN252 | 0.112 ± 0.013 abcdfg | 1.704 ± 0.076 bcd |
| AN356 | 0.113 ± 0.008 abcdfg | 1.799 ± 0.027 bcde |
| AN315 | 0.117 ± 0.003 abcdefg | 1.808 ± 0.005 bcde |
| AN242 | 0.117 ± 0.034 abcdefg | 1.814 ± 0.032 bcdef |
| AN358 | 0.120 ± 0.024 abcdefg | 1.838 ± 0.005 bcdefg |
| AN355 | 0.121 ± 0.017 abcdefgh | 1.849 ± 0.037 cdefgh |
| AN361 | 0.124 ± 0.075 abcdefghi | 1.850 ± 0.014 cdefgh |
| AN690 | 0.125 ± 0.001 abcdefghi | 1.879 ± 0.002 defgh |
| AN402 | 0.129 ± 0.000 abcdefghi | 1.981 ± 0.012 efghi |
| AN253 | 0.140 ± 0.053 abcdefghij | 2.023 ± 0.001 efghi |
| AN421 | 0.144 ± 0.031 abcdefghij | 2.046 ± 0.158 efghij |
| AN357 | 0.145 ± 0.042 abcdefghij | 2.049 ± 0.013 efghij |
| AN354 | 0.145 ± 0.047 abcdefghij | 2.049 ± 0.140 efghij |
| AN813 | 0.146 ± 0.035 abcdefghi | 2.050 ± 0.002 efghijk |
| AN332 | 0.149 ± 0.043 abcdefghij | 2.064 ± 0.004 fghijk |
| AN810 | 0.150 ± 0.049 abcdefghi | 2.075 ± 0.005 ghijk |
| AN350 | 0.154 ± 0.058 abcdefghij | 2.095 ± 0.007 hijkl |
| AN378 | 0.156 ± 0.003 abcdefghij | 2.144 ± 0.010 ijklm |
| AN704 | 0.157 ± 0.002 abcdefghij | 2.149 ± 0.005 ijklm |
| AN320 | 0.158 ±0.022 abcdefghij | 2.152 ± 0.011 ijklm |
| AN371 | 0.162 ± 0.024 abcdefghij | 2.157 ± 0.047 ijklm |
| AN250 | 0.163 ± 0.076 abcdefghij | 2.165 ± 0.035 ijklm |
| AN351 | 0.165 ± 0.055 abcdefghij | 2.197 ± 0.005 ijklm |
| AN310 | 0.168 ± 0.040 abcdefghij | 2.285 ± 0.043 jklmn |
| AN478 | 0.170 ± 0.004 abcdefghij | 2.297 ± 0.029 jklmn |
| AN419 | 0.170 ± 0.030 abcdefghij | 2.306 ± 0.004 klmn |
| AN475 | 0.172 ± 0.000 abcdefghij | 2.343 ± 0.005 lmno |
| AN496 | 0.174 ± 0.020 abcdefghij | 2.383 ± 0.067 mnop |
| AN389 | 0.175 ± 0.048 abcdefghij | 2.394 ± 0.011 mnop |
| AN346 | 0.177 ± 0.000 abcdefghij | 2.479 ± 0.042 nopq |
| AN390 | 0.180 ± 0.013 abcdefghij | 2.565 ± 0.013 opqr |
| AN249 | 0.186 ± 0.009 abcdefghij | 2.565 ± 0.004 opqr |
| AN806 | 0.186 ± 0000 abcdefghij | 2.566 ± 0.004 opqr |
| AN493 | 0.186 ± 0.016 abcdefghij | 2.576 ± 0.019 opqr |
| AN395 | 0.188 ± 0.011 abcdefghij | 2.591 ± 0.002 opqrs |
| AN382 | 0.190 ± 0.012 abcdefghij | 2.618 ± 0.018 pqrs |
| AN330 | 0.199 ± 0.031 abcdefghij | 2.624 ± 0.004 pqrst |
| AN255 | 0.203 ± 0.029 abcdefghij | 2.628 ± 0.014 pqrst |
| AN485 | 0.206 ± 0.062 abcdefghij | 2.636 ± 0.016 pqrst |
| AN487 | 0.209 ± 0.000 bcdefghij | 2.680 ± 0.007 qrstu |
| AN376 | 0.211 ± 0.007 bcdefghij | 2.704 ± 0.015 qrstu |
| AN384 | 0.218 ± 0.001 cdefghij | 2.713 ± 0.016 qrstuw |
| AN474 | 0.227 ± 0.076 defghij | 2.771 ± 0.019 rstuwx |
| AN420 | 0.229 ± 0.054 defghij | 2.776 ± 0.002 rstuwx |
| AN383 | 0.232 ± 0.055 defghij | 2.807 ± 0.003 rstuwx |
| AN484 | 0.237 ± 0.029 efhij | 2.835 ± 0.020 stuwxy |
| AN244 | 0.239 ± 0.009 efghij | 2.875 ± 0.034 tuwxyz |
| AN397 | 0.243 ± 0.008 eghij | 2.919 ± 0.020 uwxyz |
| AN472 | 0.246 ± 0.006 eghij | 2.960 ± 0.004 wxyz |
| AN247 | 0.246 ± 0.031 eghij | 2.990 ± 0.007 xyz |
| AN379 | 0.263 ± 0.016 hij | 2.997 ± 0.012 xyz |
| AN401 | 0.267 ± 0.053 ij | 3.070 ± 0.008 yz |
| AN377 | 0.278 ± 0.009 j | 3.076 ± 0.008 yz |
| AN701 | 0.283 ± 0.000 j | 3.100 ± 0.016 z |
| ***T. viridescens* complex** |  |  |
| AN328 | 0.074 ± 0.003 a | 0.946 ± 0.001 a |
| AN405 | 0.110 ± 0.038 ab | 1.410 ± 0.002 b |
| AN248 | 0.130 ± 0.042 abc | 1.451 ± 0.008 b |
| AN605 | 0.135 ± 0.003 abc | 1.485 ± 0.005 b |
| AN366 | 0.138 ± 0.026 abc | 1.689 ± 0.021 c |
| AN709 | 0.140 ± 0.001 abc | 1.970 ± 0.009 d |
| AN416 | 0.144 ± 0.022 abc | 2.064 ± 0.004 e |
| AN609 | 0.148 ± 0.002 abc | 2.191 ± 0.031 f |
| AN245 | 0.157 ± 0.007 abc | 2.284 ± 0.006 g |
| AN387 | 0.174 ± 0.036 abc | 2.399 ± 0.004 h |
| AN243 | 0.177 ± 0.033 abc | 2.446 ± 0.017 h |
| AN308 | 0.178 ± 0.074 abc | 2.619 ± 0.020 i |
| AN322 | 0.198 ± 0.015 abc | 2.662 ± 0.014 ij |
| AN334 | 0.204 ± 0.042 abc | 2.673 ± 0.005 ij |
| AN323 | 0.218 ± 0.110 abc | 2.716 ± 0.027 jk |
| AN241 | 0.226 ± 0.002 abc | 2.789 ± 0.021 k |
| AN492 | 0.227 ± 0.048 abc | 2.884 ± 0.011 l |
| AN494 | 0.236 ± 0.003 bc | 2.979 ± 0.009 m |
| AN388 | 0.260 ± 0.022 bc | 3.070 ± 0.005 n |
| AN702 | 0.271 ± 0.002 c | 3.099 ± 0.004 n |
| ***T. koningii*** |  |  |
| AN398 | 0.267 ± 0.001a | 2.001 ± 0.0077 a |
| AN399 | 0.200 ± 0.028 a | 2.052 ± 0.095 a |
| ***T. koningiopsis*** |  |  |
|  |  |  |
| AN251 | 0.166 ± 0.04 | 2.140 ± 0.162 |
| ***T. gamsii*** |  |  |
| AN327 | 0.308 ± 0.006 a | 2.463 ± 0.285 a |
| AN385 | 0.250 ± 0.034 a | 2.486 ± 0.040 a |
| ***Trichoderma* ssp.** |  |  |
| AN471 | 0.175 ± 0.01 | 2.417± 0.019 |
| ***T. reesei*** |  |  |
| QM 9414 | 0.2875 ± 0.0035 | 2.2930± 0.0066 |
